# Mapping Care Practices and Service Delivery Models for Refugee and Displaced Families in Private Hosting Arrangements: A Scoping Review

**DOI:** 10.3390/nursrep15080293

**Published:** 2025-08-11

**Authors:** Areej Al-Hamad, Yasin M. Yasin, Lujain Yasin, Andy Zhang

**Affiliations:** 1 Daphne Cockwell School of Nursing, Faculty of Community Services, Toronto Metropolitan University, Toronto, ON M5B 1Z5, Canada; lujain.yasin@torontomu.ca (L.Y.); andy.zhang@torontomu.ca (A.Z.); 2 Faculty of Nursing, University of New Brunswick, Fredericton, NB E3B 5A3, Canada; yasin.yasin@unb.ca

**Keywords:** hosting model, private hosting, refugee integration, scoping review

## Abstract

**Background/Objectives**: Private hosting arrangements have emerged as community-driven alternatives to institutional refugee housing, offering personalized support and opportunities for enhanced social integration. However, clarity around care practices and service delivery models remains underdeveloped. **Methods**: This paper presents the findings of a scoping review aimed at mapping evidence on service delivery and care practices in private hosting contexts for refugee families. Following an overview of the background and methodology, we present key themes, propose a conceptual model, and conclude with implications for policy, practice, and future research. This scoping review maps existing literature on care practices; it does not assess the effectiveness of interventions or establish best practices. The review synthesizes empirical and gray literature on service delivery and care practices supporting refugee and displaced families in private hosting contexts. Following the Joanna Briggs Institute (JBI) methodology, six academic databases and multiple gray literature sources were systematically searched, resulting in the inclusion of 28 studies. **Results**: The analysis identified four conceptual dimensions of care described in the literature: relational care and trust-building, program structure and policy integration, holistic integration pathways, and embedded equity and protection. While private hosting facilitates emotional connection and psychosocial integration, the review highlights key challenges, including variability in host preparedness, emotional labor disparities, and limited formal oversight. **Conclusions**: The findings underscore the need for evidence-informed guidelines, standardized host training, trauma-informed approaches, and coordinated policy frameworks. The resulting model offers a foundation to inform future research, guide policy development, and strengthen private hosting practices to ensure equitable, inclusive, and sustainable outcomes for refugee and displaced families.

## 1. Introduction

In recent years, private refugee hosting has emerged as a critical yet understudied component of the global refugee response. As countries continue to grapple with displacement on an unprecedented scale, formal government-led resettlement systems have increasingly been supplemented or, in some cases, supplanted by alternative models that enlist private citizens to support refugee accommodation and integration [1]. Private hosting—or private refugee sponsorship—refers to arrangements in which individuals, community groups, or organizations provide accommodation and comprehensive support (e.g., financial, social, emotional) for refugees or displaced families entering a host country, independent of traditional institutional resettlement schemes [2]. Private hosting arrangements ranging from formalized community sponsorship programs to more informal homestay placements offer a personalized and often community-embedded alternative to institutional shelter and reception systems [1,3]. Within this broad category, family-linked sponsorships represent a distinct subset, wherein sponsors have pre-existing familial ties to the refugee [4]. For example, Canada’s Private Sponsorship of Refugees (PSR) program allows family members residing in Canada to sponsor relatives abroad through family-linked pathways, while also supporting sponsorships by community groups and organizations without prior personal relationships [4]. In contrast, models like the UK’s Community Sponsorship [5] often involve private citizens or groups with no prior connection to the sponsored refugee. Private hosting encompasses a diverse range of arrangements. One well-established model, originally developed in Canada, includes private sponsorship through what are known as Sponsorship Agreement Holders (SAHs), “Groups of Five,” and community sponsors [4]. Under these streams, citizen groups commit to providing financial, social, and emotional support to refugees during their first year in the host country [4]. SAHs are formal organizations—often faith-based or community-focused—with agreements to sponsor multiple families, while Groups of Five consist of individual citizens who come together to sponsor one family, typically after refugees have already been recognized under the Refugee Convention [4]. Inspired by the Canadian model, the UK Community Sponsorship scheme was launched in 2016, where volunteer groups receive training and oversight from a lead sponsor organization and the Home Office, then support refugee families during their first one to two years [5]. This model emphasizes integration through facilitating access to housing, education, employment, and healthcare [5]. Meanwhile, homestay or charitable hosting models, such as the UK’s Refugees at Home, pair host households with individuals or families needing temporary accommodation. In these arrangements, hosts—often vetted and supported by a charity—offer lodging on a flexible, case-by-case basis [6]. By mid-2025, Refugees at Home had facilitated over 680,000 placement nights for more than 6800 refugees [6]. More recently, countries launched emergency private hosting schemes in response to crises. For example, during the 2022 Ukrainian displacement crisis, the UK’s Homes for Ukraine initiative engaged private hosts through recognized providers, combining official oversight with grassroots mobilization [7]. Finally, several countries—including Australia, Germany, Ireland, New Zealand, Argentina, and Spain—have begun replicating one or more of these models [8]. These adaptations vary in their formal structures, sponsorship duration, and integration support, but they all center community engagement in the resettlement process [8]. It is worth mentioning that these arrangements center on voluntary engagement and are typically characterized by close, domestic interactions between hosts and refugee guests [9,10].

While most hosts offer their services voluntarily, formal sponsorship programs often provide support—not payment—to facilitate host readiness. For example, Canada’s private sponsorship stipulates that sponsors must demonstrate financial capacity (through proof of income or savings) but are not compensated; however, they may receive training, pre-arrival orientation, and access to settlement service partnerships [4]. Similarly, in the UK’s Community Sponsorship scheme, volunteer groups undergo mandatory training and oversight via charitable partners and government agencies, gaining access to integration support networks [5]. Eligibility criteria typically include demonstrated capacity for financial support, English proficiency (or willingness to access language supports), and screening for safeguarding [4,5,8]. Motivations for hosts are primarily values-driven, rooted in moral obligation, empathy, community solidarity, and faith-based convictions [11]. A recent U.S. study of private sponsorship programs found that 30% of respondents cited “moral duty” and 29% “concern for family or friends,” with significantly fewer citing professional or financial incentives [11]. Such private hosting models reflect an evolving humanitarian landscape in which the responsibility for refugee care is diffused across civil society, challenging conventional state-centric approaches to resettlement [12,13].

A growing body of research suggests that private hosting can significantly enhance integration outcomes, particularly in the domains of social connection, psychological well-being, and everyday navigation of local systems [7,10,14]. Herpell, Marbach [14], for example, report statistically significant improvements in these dimensions among Ukrainian refugees in the UK hosted through the “Homes for Ukraine” scheme, where participants shared living spaces with their hosts, engaged in frequent interpersonal interactions, and received assistance with essential tasks such as job searching and accessing healthcare. Similarly, Dauphin and Veronis [15] found that Syrian refugees supported through private sponsorship programs in Canada experienced more diverse, sustained, and valued forms of support compared to those resettled through government-led pathways. These supports often facilitated stronger social networks, improved housing stability, and a deeper sense of belonging within local communities [1,9]. In Canada, the principle of “additionality” is central to this balance: private sponsorship is intended to complement rather than replace government-assisted resettlement quotas [16]. However, in other contexts, concerns have emerged about governments relying too heavily on private initiatives to fill systemic gaps in refugee protection [16].

The private hosting model varies widely in host readiness and motivation, shaping the quality of support and emotional dynamics; while some foster empowering environments, others may unintentionally reinforce power imbalances or limit refugee autonomy through imposed cultural expectations [1,17]. The decision by private citizens to host refugees is often driven by altruistic and moral motivations, including a sense of social justice, religious or ethical convictions, and a desire to foster community solidarity [11]. While most private hosts do not receive financial compensation, many programs provide training, logistical support, and emotional preparation to help mitigate the personal, financial, and emotional costs of hosting [11]. However, concerns about the sustainability of such reliance on private actors have been raised, particularly in contexts where hosts may lack ongoing institutional support. This highlights the need for policies that balance community engagement with structural safeguards to prevent burnout and inequities [11].

Many of the identified hosting practices—such as trauma-informed and culturally responsive approaches, strengths-based and family-centered methods, and services like case management and psychosocial support—are primarily implemented by professional social service agencies [4,5,8,11]. However, in some private hosting programs, hosts are provided with training and support to incorporate these practices at the household level [11]. For example, Canada’s Private Sponsorship of Refugees program includes orientation and settlement supports designed to prepare sponsors for culturally responsive and trauma-informed engagement [4], ensuring these efforts complement, rather than replace, professional services in line with the principle of “additionality” [16]. The interpersonal nature of hosting, while intimate and relational, can also become a site of emotional labor, boundary negotiations, and latent tensions, particularly when support structures for hosts themselves are lacking [1,9]. Furthermore, the reliance on private citizens to provide essential settlement services raises broader questions about the shifting responsibilities of the state, the sustainability of such arrangements, and the risk of normalizing a two-tiered system of refugee support [7,13]. The term “two-tiered system of refugee support” refers to disparities in access to and quality of assistance based on refugee characteristics such as nationality, religion, and legal status [7]. Studies have noted that refugees perceived as white, Christian, or European are often prioritized in private hosting and sponsorship programs, reflecting broader patterns of racialized and geopolitical bias in resettlement systems Importantly, the privileging of certain refugee groups such as white, Christian, or European-identifying individuals within private hosting schemes underscores a pattern of racialized selectivity and humanitarian conditionality [7]. This does not imply that minority citizens or non-Christian organizations are unwilling to participate as hosts, but rather highlights systemic factors shaping which refugees are more likely to be selected [7]. These patterns suggest that without safeguards, private hosting risks reproducing existing structural inequalities and exclusions [7]. For example, Burrell [7] notes that Ukrainian refugees have received preferential treatment in hosting and sponsorship schemes compared to non-European asylum seekers, reflecting broader geopolitical hierarchies of empathy and deservingness. These dynamics reveal the ways in which private hosting, while potentially transformative, can also reproduce structural inequalities and perpetuate exclusions grounded in race, class, religion, and legal status [7].

While some studies have evaluated the outcomes of private sponsorship programs and examined host perspectives, there remains a notable gap in understanding the relational, emotional, and political dimensions of private hosting from a critical, intersectional perspective [18,19]. Much of the literature focuses on measurable indicators of integration, such as employment or housing, while underexploring how hosts and refugees co-construct notions of care, belonging, and responsibility in shared domestic spaces [1]. Moreover, few studies explicitly engage with the perspectives of women and racialized migrants navigating these arrangements, nor do they fully examine how gendered and cultural expectations shape hosting relationships and influence long-term integration trajectories [1,19]. Kulovic [20] argues that sustainable integration depends on broadening the scope of private sector involvement beyond market-driven opportunities to include meaningful engagement with refugee needs and host community development. This entails a shift from viewing refugees as passive recipients of aid or isolated economic agents to recognizing them as central stakeholders in co-developing inclusive systems that support self-reliance and communal resilience [20]. A multi-stakeholder approach that integrates private actors into institutional frameworks while holding them accountable to equity-driven outcomes offers a pathway toward more effective and ethical refugee integration strategies [7,10,19].

These debates are mirrored in policy discussions surrounding private refugee sponsorship, particularly in Western contexts such as Canada and Europe. Although private sponsorship is often lauded for its potential to enhance refugee resettlement by mobilizing community support and social capital [21] tensions arise when the goals of private sponsors clash with those of the state. In Canada, the principle of “additionality”—the idea that private sponsorship should supplement, rather than replace, government resettlement—has become increasingly contested as administrative burdens and shifting regulations threaten sponsor capacity and, by extension, overall resettlement targets [22]. In the European context, ref. [23] advocates for carefully designed private sponsorship models that respect state sovereignty while leveraging the compassion and initiative of private citizens. Effective implementation requires balancing security, integration, and welfare objectives with the humanitarian imperatives driving sponsorship initiatives [22,23]. Family-linked sponsorships, in particular, reveal the deeply emotional and personal motivations that fuel many private hosting efforts, especially as former refugees transition into sponsor roles themselves [21]. However, these intimate ties also underscore ongoing challenges related to family separation, emotional strain, and gaps in formal state support [21].

While private hosting has gained attention during major global displacement crises, including those involving Syrian, Afghan, and Ukrainian refugees, there remains limited synthesis of how care practices and service delivery models function in these arrangements. Existing research is fragmented, with few studies providing mapping of the evidence or considering diverse geographic and sociopolitical contexts. These knowledge gaps underscore the need for a comprehensive mapping of the evidence base to inform future practice, policy development, and the design of equitable support systems for refugee and displaced families. Addressing the identified literature gaps is essential for advancing the understanding of care practices and service delivery within private hosting arrangements. Similarly, the predominant focus on quantifiable integration outcomes, such as employment and housing, neglects relational and process-oriented aspects of care—such as trust-building, cultural safety, and psychosocial well-being—that are critical to holistic and equitable service delivery [4]. Filling these gaps supports the identification of promising models that are both inclusive and contextually responsive.

Collectively, these insights emphasize the need for coordinated and equity-focused private hosting models to foster refugee integration—one that recognizes the economic interdependence of refugees and hosts, supports ethical engagement of the private sector, and fosters collaboration rather than conflict between state and non-state actors [21,22,23]. As private hosting and sponsorship continue to evolve, it is essential to ensure that these models are not only effective in supporting refugee well-being but are also grounded in inclusive, just, and sustainable frameworks of care. This scoping review maps heterogeneous evidence on care practices and service models for refugee and displaced families in private hosting, highlighting emerging patterns across diverse contexts and identifying gaps to inform future models and practice guidelines. This scoping review aims to answer the following question: “What care practices and service delivery models are identified in the literature to support refugee and displaced families in private hosting arrangements?”.

## 2. Materials and Methods

This scoping review followed the Joanna Briggs Institute (JBI) methodology [24] was guided by the Preferred Reporting Items for Systematic Reviews and Meta-Analyses extension for Scoping Reviews (PRISMA-ScR) guidelines [25]. In line with JBI guidance, we consulted key stakeholders, including policymakers and service providers, to validate findings, contextualize results, and enhance the analytical validity of this review [26]. The protocol was registered at Open Science Framework (OSF): Registration DOI https://doi.org/10.17605/OSF.IO/NC2TK (accessed on 19 July 2025).

### 2.1. Inclusion and Exclusion Criteria

This scoping review was guided by the JBI Population, Concept, and Context (PCC) framework [24] which provided a structured approach to clearly define the target population, the key concept of interest, and the relevant contextual settings. Inclusion criteria comprised peer-reviewed and gray literature of all study types (qualitative, quantitative, mixed-methods, and reviews), published in English from January 2000–April 2025 to capture the evolution of private hosting models in response to major global displacement crises, including those involving Syrian, Afghan, and Ukrainian refugees. This period reflects significant developments in care practices and service delivery for refugee and displaced families in diverse contexts. Studies focusing exclusively on refugees in institutional or camp settings were excluded. A detailed breakdown of these criteria is provided in Table 1.

### 2.2. Search Strategy

The search strategy was developed in collaboration with a research librarian and incorporated a broad range of relevant keywords and indexing terms, initially tailored for the CINAHL database, as outlined in Supplementary S1. It was subsequently adapted for use across other databases, taking into account differences in Boolean operators, truncation, and wildcard symbols. A second librarian conducted a peer review of the strategy using the PRESS (Peer Review of Electronic Search Strategies) guidelines [27]. The search targeted English-language publications from 2000 to 2025, and reference lists of included studies were screened to identify additional relevant sources. The starting point of 2000 was selected due to significant policy developments and a global increase in displacement, which contributed to a growing body of research on refugee hosting and settlement. This timeframe allowed for the inclusion of current and contextually relevant literature. The databases searched included CINAHL (*n* = 127), Medline (*n* = 190), SocINDEX (*n* = 2), Scopus (*n* = 10), Web of Science (*n* = 8), and ProQuest Dissertations and Theses (*n* = 1). Additional searches were conducted using Google Scholar and manual screening of reference lists (*n* = 26). Gray literature, including conference abstracts and opinion papers, was also considered to ensure a comprehensive and inclusive review [28].

### 2.3. Study Selection and Extraction

**Figure 1:**
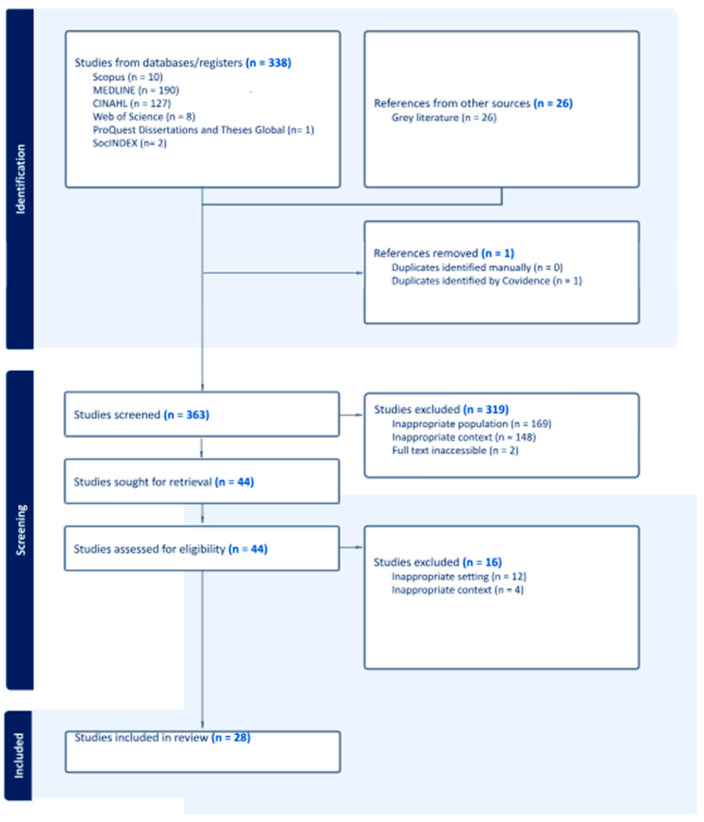
PRISMA diagram for selected studies.

Following the completion of the search, all identified records were consolidated and imported into EndNote v.21, where duplicate entries were systematically removed. Two reviewers (AZ and LY) independently conducted an initial screening of titles and abstracts to assess alignment with the predetermined inclusion criteria. Articles deemed potentially relevant were retrieved in full, and citation details were imported into Covidence for streamlined organization and analysis. A second round of title and abstract screening was conducted using the same inclusion criteria, followed by a comprehensive full-text review of eligible studies. For each study excluded at this stage, the rationale for exclusion was documented. Disagreements between reviewers were addressed through discussion or by involving a third reviewer (YY) for consensus. The entire screening and selection process was captured in a PRISMA flow diagram (Figure 1). Data from the final selection of studies were extracted using a customized chart based on a modified version of the standardized JBI data extraction tool (Supplementary S2), tailored to address the specific focus of the review [29]. Extracted information included participant characteristics, study concepts, contextual details, methodologies, and major findings. Any inconsistencies in data extraction were resolved collaboratively, with arbitration from a third reviewer (YY) when necessary. In cases of incomplete or unclear data, study authors were contacted for clarification [24].

### 2.4. Data Analysis

The data analysis followed a foundational inductive content analysis methodology [30] in which two reviewers (A.Z. and L.Y.) independently recorded the key characteristics of the included sources and tracked how often various aspects of refugee private hosting, such as barriers, strategies, and outcomes, were addressed. The reviewers thoroughly analyzed a range of materials, including peer-reviewed articles, reports, and gray literature, to identify recurring codes linked to the study’s target population, core concept, and contextual settings. These initial codes were then clustered into broader thematic categories by identifying emerging patterns, convergences, and divergences across the data. This analytic process was dynamic and iterative, allowing for ongoing adjustments as new insights emerged during review [30]. To enhance the rigor and reliability of the findings, the team adopted a comparative method to address any inconsistencies, resolving them through dialog or involving a third reviewer (Y.Y.) when necessary. The final coding structure and extracted data are presented in Supplementary S2.

## 3. Results

The initial search identified 338 records from the searched databases and 26 records from gray literature. After eliminating one duplicate and removing any entries due to incomplete information, 363 records were retained for initial screening. Titles and abstracts were reviewed to assess alignment with the study’s aims, narrowing the selection to 44 articles for full-text review. Of these, four were excluded due to contextual irrelevance, and 12 were deemed ineligible based on context. No additional records were identified through targeted searches on Google Scholar and by manually reviewing reference lists of included studies. In total, 28 records met the inclusion criteria and were incorporated into the final scoping review. This review maps existing literature and does not evaluate study quality or intervention effectiveness. The full selection process is depicted in the PRISMA flow diagram (Figure 1).

### 3.1. Description of Studies

The included studies reflect a diverse geographical spread, offering a range of insights into private hosting experiences for refugee and displaced families across various regional contexts. Studies were conducted in the United Kingdom [7,31,32,33], Germany [14,34], Jordan [35], Slovakia [10], France, Britain, and Italy [36], Canada [1,9,37,38,39,40,41,42], the Democratic Republic of the Congo [43], and the Occupied Palestinian Territories [44]. Broader regional analyses included countries across Europe, and global or multi-country perspectives were presented in studies with a worldwide or pan-European scope [45,46]. Methodologically, the review encompassed a range of qualitative designs [1,7,10,14,33,35,36,43]. Quantitative approaches included surveys and quasi-experimental designs [10,14]. Four reviews [31,44,47,48] and one concept analysis [3] were also included. Additionally, one study used mixed methods [10] and 11 reports and newsletters articles [2,37,38,39,40,41,43,45,46,49,50,51,52]. This variety in geographical and methodological focus enriches the review’s understanding of private hosting experiences for refugees and displaced families’ practices across different sociopolitical contexts. The following discussion presents four interrelated dimensions that emerged across the literature: (1) relational care and trust-building, (2) policy and program structure, (3) integration pathways, and (4) equity and protection.

### 3.2. Relational Care and Trust-Building as Core to Effective Hosting (Interpersonal Dimension)

The literature describes care practices as involving mutual respect, empathy, and relational reciprocity. Mutual respect involves recognizing and considering each other’s inherent value and contributions, and is necessary for peaceful coexistence in the hosting relationship [3]. Respect from hosts entails not only offering a warm welcome but also acknowledging and valuing the guests’ lived experiences, perspectives, and individual needs [7]. In turn, guests are expected to reciprocate by appreciating the host’s generosity and demonstrating regard for their household routines, cultural norms, and personal boundaries [36]. Empathy is a key motivator for hosting behaviors [43]. Often, hosts are driven by a sense of compassion and willingness to help, stemming from personal experiences with displacement [43]. Empathy encourages mutual understanding and perspective-taking [10] which is important to navigate differences that may impact the hosting relationship and allow individuals to attune to the needs of others. Caring in the host relationship thus shifts from the perspective of “caring for” to “care about” the other [35]. Relational reciprocity shifts the hosting relationship from a one-directional act of giving to a shared, mutual exchange of support and care based on shared respect and hospitality [35].

Successful hosting arrangements foster trust and emotional connection, often developing into familial bonds [3]. Trust serves as the cornerstone of a positive and meaningful hosting relationship. For refugees, stepping into the home of a stranger often entails a profound sense of vulnerability and uncertainty, which can complicate their capacity to adapt to a new and unfamiliar environment [36]. Establishing trust not only helps mitigate these challenges but also creates a sense of emotional safety, enabling refugees to engage more openly and confidently within the hosting arrangement [49]. For hosts, opening their personal space to others demands a significant degree of openness, adaptability, and a readiness to share elements of their daily lives and personal boundaries [7]. Building trust from the outset of the hosting experience is essential, as it lays the groundwork for mutual understanding, emotional safety, and a genuine sense of belonging [33]. Early trust-building fosters shared intimacy, transforming the hosting arrangement from a temporary shelter into a space of meaningful human connection [33]. Hosts who offer early gestures of welcome present a positive first impression, set a welcoming tone for the hosting experience, and ultimately aid in building trust in the initial stages of the hosting arrangement [33]. Once trust and emotional connection are established, hosting relationships may flourish into a ‘like-family’ dynamic where refugees and hosts recognize one another as family [32,33,34,36]. This relational shift is represented through sharing household responsibilities, symbolic inclusion of the refugee in the household, such as sharing photographs, nicknames, meals, and use of familial titles ‘like a sister’ as terms of endearment [33]. These familial bonds lead to positive refugee outcomes, including facilitating social integration and a sense of belonging [33]. Refugees and hosts often viewed this family relationship as one that endures over time regardless of any changes in circumstances [33,34], emphasizing the importance of hosting relationships in building a strong sense of lasting kinship that may aid in the resettlement process.

Studies report that hosting arrangements involve a balanced sharing of care responsibilities, with both hosts and guests contributing meaningfully [47]. While hosts often provide space, resources, and support, this role can lead to physical, emotional, social, and financial strain if adequate support is lacking [47]. This may further contribute to the negative discourse of refugees as a burden to the hosting system [47]. Guests also have the capacity to contribute in ways that affirm their presence as active participants in the hosting relationship rather than passive recipients of support [7]. Reconfiguring hosting arrangements to one that emphasizes shared responsibilities can help to alleviate the burden on hosts and ensure the sustainability of the hosting relationship in the long term [7,10,35]. However, without clearly defined boundaries and equitable relational dynamics, emotional strain, particularly for women, can emerge within hosting arrangements [3]. Research on the relational and affective dimensions of hosting highlights the substantial emotional labor involved in sustaining these relationships, as hosts often navigate complex feelings of responsibility, empathy, and social expectation to ensure the arrangement remains stable and supportive [7,36]. Hosting responsibilities often fall disproportionately on women due to a domestic responsibility towards caregiving, placing them at risk for additional emotional burden [7].

### 3.3. Program Structure, Support Systems, and Policy Integration (Structural Dimension)

High-quality private hosting depends on well-structured programs with defined roles, expectations, and safeguards for both hosts and guests. The absence of such frameworks places undue burden on hosts and limits guests’ well-being, underscoring the need for clear practices [1]. Humanitarian actors supporting hosting relationships should clearly define minimum standards for hosting, such as a list of goods and services, so there are shared expectations in the hosting arrangement [47]. Formal specialized training is required to equip hosts with the necessary knowledge and skills to properly support refugees during the hosting experience and resettlement journey [14]. Clear guidelines are needed to regulate private hosting, including screening, verification, and monitoring processes to protect guests and support hosts, and should involve minimum housing standards, vetting procedures, and safeguards against exploitation [14,45]. Strategies include formal matching services, government-supervised matching procedures, pre-screening hosts, background checks, formal interviews, on-site inspections, and housing visits [14,45]. Information should be provided to refugees that outlines available services, housing options, and legal rights to support the resettlement process [14,45].

Access to support services should be readily available to aid in maintaining emotional, psychological, and social well-being during hosting [47]. Although hosting arrangements are often perceived to be a temporary assignment, this is often not the reality [46]. Hosting arrangements must contain clear exit strategies that allow refugees to transition from hosting arrangements into permanent housing [46]. Sustaining private hosting requires coordinated policy support and government accountability to promote equity across communities [38]. With rising numbers of refugees and migrants globally, there is a growing demand for temporary housing, yet resources and infrastructure remain limited [1,38]. Although private hosting arrangements are known to provide essential housing and integration opportunities for newly arrived individuals in times of crisis, there is currently no sustainable private housing framework in place to support the long-term outcomes of these vulnerable individuals [1,46]. Enhanced coordination among civil society, humanitarian organizations, government agencies, and volunteer hosting networks is essential to strengthen private hosting programs [45,46]. Such collaboration can improve program effectiveness, ensure consistent support, and facilitate smoother transitions toward long-term resettlement for at-risk refugees [45,46].

### 3.4. Holistic Integration Pathways (Integration Dimension)

Private and homestay hosting have emerged as powerful, human-centered approaches to refugee integration by fostering close, everyday relationships that support emotional healing, build trust, and encourage a sense of inclusion [1,7,10]. Programs such as Room for Refugees and Refugees Welcome International show how guests often grow to feel included in their host families, benefiting from informal support with language, navigation of local systems, and emotional stability [50,51]. These relationships extend beyond basic hospitality to include shared meals, mutual cultural and language learning, and the development of personal autonomy, consequently improving well-being and confidence among refugees while allowing hosts to feel fulfilled and more civically engaged [1,9,32,33]. However, the dynamics within these hosting arrangements are often complex, shaped by intersecting social, cultural, and emotional factors that influence daily interactions and relationships [9].

The unequal distribution of emotional labor often falls on women, and the pressure on refugees to conform to host expectations can subtly reinforce social hierarchies which ultimately depend on the host-refugee relationship [34]. While personalized care in private hosting can be transformative, overreliance on individuals to support refugees highlights critical gaps in systemic infrastructure [1,10]. Many hosts report feeling overwhelmed by the emotional and logistical burdens, particularly when state support is limited or inconsistent [7]. Refugees, in turn, can feel abandoned by formal institutions, especially when faced with uncertain housing futures or transition anxiety without proper planning [46]. Although private hospitality can craft a foundation for inclusion, domestic environments are not automatically safe or equitable, and risks such as dependency, exploitation, or cultural miscommunication must still be actively addressed [1,2,42]. To ensure equitable outcomes, integration plans must adapt to the nature of each hosting arrangement, including familial, host-based, or institutional, and be supported by wraparound services such as language training, employment pathways, and legal aid [1,9].

Effective private hosting arrangements are shaped by a variety of contextual and interpersonal factors that influence how well refugees integrate socially, psychologically, and navigationally [47]. Caron [47] identified key environmental factors such as relationship type, length of stay, and shared living spaces that shape hosting dynamics and influence trust and emotional connection. Sharing daily life can foster cultural exchange and, when combined with supports like language or job assistance, significantly improve integration outcomes [47]. Furthermore, Herpell, Marbach [14] found that private hosting fosters stronger social and emotional integration than public shelter systems, even though its impact on economic and language outcomes is more limited and underscoring the distinct value of relational immersion. Hosting models that reflect hospitality and shared humanity, especially those that regard refugees as “family”, can be profoundly transformative for both guests and hosts [36]. Monforte, Maestri [36] found that volunteers often hosted out of moral duty and affection, viewing guests as “new family members”. While this fosters empathy and emotional healing, it can also create tension if refugees are seen as ungrateful, highlighting the need for clear boundaries and mutual understanding [36]. Similarly, Al-Hamad, Yasin [3] highlight the importance of balancing hospitality with power dynamics and cultural sensitivity. Their model emphasizes that successful cohabitation relies on conditions such as mutual respect, peaceful coexistence, and therapeutic presence [3]. To ensure equitable outcomes, safe practices in private hosting must also include structural supports such as host training, dispute resolution mechanisms, and minimum standards for care, aligning emotional warmth with practical stability [47].

### 3.5. Embedding Equity, Protection, and Accountability in Hosting Design (Protection Dimension)

While private hosting initiatives have proven success in offering immediate shelter and emotional support to displaced persons, their design often lacks structural protections essential to uphold safety, dignity, and equity for women, children, and other vulnerable groups [1,42]. Without formal oversight, hosting arrangements risk reinforcing systemic inequalities, despite being framed as acts of humanitarian care [7]. Hosts, especially women, frequently shoulder disproportionate emotional and bureaucratic burdens, while guests experience feelings of abandonment and instability [34]. These risks are compounded by assumptions that domestic spaces are inherently safe, overlooking the power imbalances, emotional dependencies, and cultural misunderstandings that can emerge in unregulated environments [2,42].

Effective models like buddy schemes and the Room for Refugees program, which has provided over half a million shelter nights across the UK, Ireland, and the U.S., show that mutual support and cultural exchange can promote meaningful inclusion, but only with clear expectations and structured support [32,51]. Embedding equity and protection at the core of hosting design is not optional; it is essential to ensure that the burden of care does not shift unfairly onto individuals and that guests are not left navigating fragile systems alone [46,50].

To meaningfully embed equity and protection in refugee hosting, hosting arrangements must move beyond informal goodwill and instead be underpinned by formalized structures that prioritize safeguarding and mutual accountability [2]. Caron [47] stresses that private hosting, while potentially beneficial, requires systematic oversight to prevent risks such as exploitation, overcrowding, and emotional dependency. The design of hosting programs must therefore include core elements such as minimum standards of living, proactive dispute resolution mechanisms, and clear guidelines for shared responsibilities [39]. These critical elements, including host screening, housing inspections, and criminal background checks, can create safer environments and also empower hosts by providing clarity, reducing miscommunication, and setting expectations for respectful cohabitation [39].

Embedding equity also involves recognizing and addressing the underlying power dynamics that shape host-guest relationships [9,34]. As Al-Hamad, M. [1] notes, hosting is shaped by values and can either promote inclusion or reinforce inequalities tied to gender, class, or culture. To address this, programs must include cultural competence training focused on mutual respect, trauma-informed care, and cross-cultural communication [1,9]. While many hosts may be driven by affective responsibility and a moral desire to help, this can unintentionally lead to exclusionary attitudes toward refugees who do not conform to perceived norms of gratitude or emotional reciprocity [36]. Thus, equity in hosting design must include not only logistical and material considerations but also a social and emotional framework that promotes mutual dignity [2,52].

## 4. Discussion

**Figure 2:**
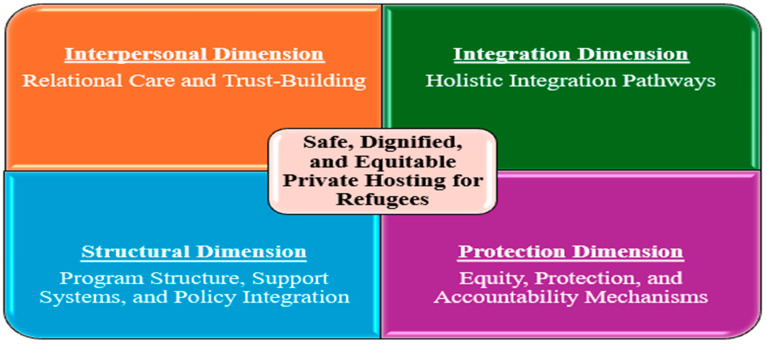
Dimensions of a safe, dignified, and equitable private hosting model for refugees.

This scoping review aimed to examine existing care practices, service delivery models, and support mechanisms for refugee and displaced families living in private hosting arrangements. This review maps existing literature and does not evaluate study quality or intervention effectiveness. The analysis revealed several key themes, including the critical role of host-guest relationships in shaping psychosocial integration, the emotional labor and boundary negotiations involved in hosting, and the impact of structural gaps on both host and refugee experiences. While private hosting offers intimate, community-based support with strong relational potential, the review also highlighted inconsistencies in host preparedness [18,32], limited formal oversight [9], and the need for coordinated, equity-oriented frameworks [19,22,23]. These findings underscore both the promise and the challenges of private hosting as an alternative model of refugee accommodation. The review’s findings informed the development of a conceptual model (See Figure 2) for safe, dignified, and equitable private posting for refugees, highlighting four intersecting dimensions: Interpersonal, Structural, Integration, and Protection. These dimensions offer a holistic framework for understanding the relational, systemic, and ethical components that shape private hosting experiences. Each dimension draws on empirical and conceptual insights from the literature, underscoring care practices while also exposing critical gaps in design, delivery, and oversight. The following discussion outlines each dimension and positions the findings within broader scholarly and policy debates.

The review found that mutual respect, empathy, and relational reciprocity are foundational to effective hosting, shaping trust, emotional safety, and eventual kin-like relationships between hosts and refugees [9]. This finding resonates with studies by Monforte, Maestri [36] and Sirriyeh [33] who similarly highlight how emotional connection and everyday intimacy can transform hosting into a deeply humanizing and empowering experience. Consistent with Canter [32], the review emphasizes how symbolic gestures like shared meals or familial nicknames can foster belonging. However, the review also underscores a key tension: without clear boundaries, emotional strain, particularly for women, can emerge, echoing findings by [1,7] and reinforcing Stock’s [34] concerns about gendered caregiving burdens. This aligns with Caron’s [47] warning that unsustainable expectations placed on hosts, especially women, can lead to burnout and undermine the longevity of hosting relationships.

The review identified the critical need for structured programs with clear guidelines, screening protocols, host training, and formal support to ensure the safety and sustainability of hosting arrangements. These findings are strongly supported by Herpell, Marbach [14], and Organization for Economic Co-operation and Development (OECD) [45], who advocate for formal matching, vetting, and monitoring mechanisms to standardize quality and reduce risks. Caron [47] similarly underscores the importance of clearly defined expectations and support structures. Consistent with the International Federation of Red Cross and Red Crescent Societies (IFRC) [46], the review emphasizes the need for exit strategies to prevent prolonged dependency and ensure smooth transitions to permanent housing. These findings reaffirm Kulovic’s [20] call for multi-stakeholder engagement and Keung’s [38] observation that rising refugee arrivals outpace existing infrastructure, demanding coordinated policy responses. However, while literature calls for more sustainable frameworks, few models offer scalable, equity-centered approaches, indicating a continued gap in translating emerging practices into policy.

The review affirms that private hosting enhances social, emotional, and navigational integration through close, trust-based relationships that foster personal growth, cultural exchange, and informal mentorship [1,10,50]. These findings are consistent with Caron [47] and Herpell, Marbach [14], who note that social and psychological integration outcomes are stronger in private hosting than in public shelter systems. Programs like Room for Refugees [51] and Refugees Welcome [50] show how integration thrives when guests feel included in household routines. However, consistent with Stock [9,34], the review highlights power asymmetries, gendered labor expectations, and emotional overinvestment as barriers to equitable integration. These tensions suggest that while private hosting can foster deep inclusion, it must be paired with wraparound support such as legal aid, employment pathways, and language training to avoid perpetuating inequities and dependence [1,19].

The review findings emphasized that while private hosting provides immediate shelter and emotional support, it often lacks formal protection for vulnerable populations. This insight is consistent with several studies [7,41,46], all of whom stress that private arrangements may reproduce systemic inequalities if left unregulated. Emotional dependencies, misaligned expectations, and assumptions about domestic safety can result in exploitation, burnout, or neglect [9,34]. The review supports Caron’s [47] and Operation Ukrainian Safe Haven’s [39] advocacy for minimum living standards, host screening, and dispute resolution as essential components of hosting design. Furthermore, the review aligns with Al-Hamad et al. [1] and Monforte et al. [36] in calling for cultural competence training to address underlying power dynamics and to prevent exclusionary practices. These insights underline the need to embed equity into the structural, social, and emotional fabric of hosting to ensure safe, dignified, and rights-based support for refugees.

Following the JBI’s recommendations for engaging knowledge users in scoping reviews [26], we shared our preliminary findings with six key stakeholders to assess the relevance and resonance of our results in relation to their firsthand experiences with refugee private hosting models. This engagement aimed to validate our interpretations and ensure the findings reflected practical realities on the ground [26]. Faculty members with scoping reviews experience (*n* = 2), Executive Directors of non-profit organizations working with refugee settlement (*n* = 2), and faculty members with research programs in refugee settlement and migration (*n* = 2). Stakeholders were asked to review our results and suggest topics for discussion and future research. All six stakeholders agreed that our findings corresponded with their experiences.

### 4.1. Implications

This review offers several important implications for practice, policy, and research related to private hosting of refugee and displaced families. In practice, effective private hosting requires clear role definitions, adequate training, and sustained psychosocial support for both hosts and guests. Many hosting initiatives rely on the goodwill of individuals, often without sufficient guidance or institutional backing, which can lead to emotional exhaustion, especially among women hosts who disproportionately shoulder caregiving responsibilities [1,7]. Care practices are shaped by context-specific factors like institutional support or host training. Programs must offer trauma-informed training that prepares hosts for the emotional and cultural complexities of refugee support and equips them to navigate boundary-setting, communication, and emotional labor [9,10]. Additionally, wraparound services including mental health support, language instruction, legal assistance, and job-readiness programs should be integrated into hosting arrangements to ensure comprehensive care and promote the long-term well-being and autonomy of guests [10,47].

From a policy perspective, the findings underscore the urgent need for coordinated, state-supported frameworks to regulate and sustain private hosting as a formal resettlement mechanism. Currently, many hosting arrangements operate in legal and operational gray zones, leaving both hosts and refugees vulnerable to exploitation, instability, and burnout [45,46]. Policymakers should develop standardized procedures for host screening, housing verification, grievance redress, and minimum care standards to ensure safety and equity in hosting environments [39]. Integration of private hosting within broader national housing and migration strategies, such as housing subsidies, resettlement quotas, and exit pathways to permanent housing, can strengthen system responsiveness and support continuity of care beyond the hosting period [38]. Moreover, policies must be equity-responsive, recognizing how intersecting factors like gender, race, class, and immigration status shape the experiences of both hosts and guests. Embedding cultural competence training and equity principles in hosting programs can help mitigate relational power imbalances and improve intercultural understanding [1,36]. Findings suggest potential avenues for policy innovation, such as standardized training modules for hosts grounded in cultural humility and trauma-informed care.

Despite a growing body of literature, substantial gaps remain in understanding the long-term impacts of private hosting on refugee well-being and host communities. Future research should explore longitudinal outcomes for hosted refugees in areas such as housing stability, employment integration, mental health, and social belonging. Comparative studies across national contexts are also needed to evaluate different hosting models such as familial, volunteer-based, or faith-based arrangements and their relative effectiveness in promoting integration and equity. Importantly, more research is required to understand hosts’ motivations for participating in private hosting initiatives. While some are driven by empathy, moral responsibility, or shared cultural or religious identities [36,43], others may be influenced by social capital, ideological commitments, or economic incentives. Understanding these motivations can help shape recruitment strategies, host retention, and targeted training interventions. In addition, further inquiry is needed into how private refugee hosting is advocated and communicated in public discourse and policymaking. This includes examining the role of civil society, media narratives, and government messaging in framing hosting as a civic duty, humanitarian response, or integration strategy. Finally, future studies should adopt participatory and co-design approaches that prioritize the lived experiences of both hosts and refugees in the development and evaluation of hosting programs. Evaluating the impact of formal policy interventions such as host subsidies, training mandates, or legal protections will be crucial for informing scalable, equitable models that can be adapted to diverse sociopolitical contexts.

### 4.2. Strengths and Limitations

A key strength of this scoping review is its comprehensive and methodologically rigorous approach, guided by the JBI framework and PRISMA-ScR guidelines. The inclusion of peer-reviewed and gray literature from a wide range of geographical and cultural contexts enhances the breadth and relevance of findings. The review includes a significant proportion of gray literature, which is commendable for inclusivity but may introduce reporting bias—particularly when sources originate from organizations involved in implementing the programs under study. Organizational sources may be more likely to emphasize program successes while underreporting challenges. To enhance the integration of gray literature and to mitigate any potential biases, gray literature was reviewed for potential bias by analyzing institutional affiliations and funding sources. Findings were triangulated with academic studies to assess convergence and divergence in reporting. The engagement of stakeholders further strengthened the applicability and resonance of the results. However, the review is limited to English-language publications, which may exclude valuable insights from non-English speaking regions with relevant hosting models. Additionally, while gray literature was included, variability in the depth and quality of reporting across sources may have impacted the completeness of extracted data. The heterogeneity of hosting contexts also poses challenges in drawing universally applicable conclusions.

## 5. Conclusions

Private hosting represents a promising, human-centered approach to refugee accommodation, capable of fostering meaningful inclusion, emotional healing, and community engagement. However, to be sustainable and equitable, hosting arrangements must be supported by formalized structures, policies, and services that address systemic inequalities and protect the dignity of all participants. This review identifies key dimensions of safe, dignified, and equitable private hosting for refugees, including relational, structural, integrative, and protective aspects that should inform future program design and policy development. Embedding equity, cultural sensitivity, and accountability in hosting practices will be essential for transforming hospitality from an individual act of goodwill into a stable, rights-based mechanism for refugee support.

## Figures and Tables

**Table 1:** Inclusion and Exclusion Criteria.

Criteria	Inclusion	Exclusion
Literature Type	Peer-reviewed and gray literature (e.g., UNHCR, NGOs, government reports), including commentaries, editorials, opinion pieces, websites, newspapers, and letters to the editor.	Duplicate sources or retracted publications and literature that are not publicly accessible.
Study Type	All study types (qualitative, quantitative, mixed-methods studies were included only if findings from private hosting could be disaggregated and various forms of relevant reviews).	Duplicate studies of the same study without new or additional analysis or incomplete conference abstracts.
Language	English.	Non-English publications
Publication Date	January 2000–April 2025.	Published before January 2000 or after April 2025.
Population	Refugee and displaced families (including asylum seekers, stateless individuals, and those forcibly displaced due to conflict or disaster).	Populations not identified as refugee/displaced or lacking clarity on displacement status.
Context	Private hosting arrangements (i.e., settings where refugee or displaced families are accommodated within private homes).	Institutional settings (e.g., camps, shelters, group homes).
Concept	Care practices and service delivery models considered “care practices,”	Care practices and service delivery models that do not focus on refugee or displaced populations in the context of private hosting.

## Data Availability

All data generated or analyzed during this study are included in this article.

## Supplementary Materials

The following are available online at https://www.mdpi.com/article/10.3390/nursrep15080293/s1, Supplementary S1: Search Strategy; Supplementary S2: Extraction Table.

## Public Involvement Statement

No public involvement in any aspect of this research.

## Guidelines and Standards Statement

This manuscript was drafted against the Preferred Reporting Items for Systematic Reviews and Meta-Analyses Extension for Scoping Reviews (PRISMA-ScR).

## Abbreviations

The following abbreviations are used in this manuscript:

JBI	Joanna Briggs Institute
(PRISMA-ScR)	Systematic Reviews and Meta-Analyses extension for Scoping Reviews
PCC	Population, Concept, and Context
