# Supplementary material for: Mapping Care Practices and Service Delivery Models for Refugee and Displaced Families in Private Hosting Arrangements: A Scoping Review
Source: Nurs Rep. 2025 Aug 11;15(8):293. doi: 10.3390/nursrep15080293 (PMC12389609; doi:10.3390/nursrep15080293)
Supplement: Supplementary file 1 [file nursrep-15-00293-s001.zip › Supplementary S1.pdf]

Supplementary S1: Search Strategy

Database	Search strategy
MEDLINE (Ovid)	1- exp Refugees/ or refugee*.tw,kf. or asylum seeker*.tw,kf. or displaced famil*.tw,kf. or displaced person*.tw,kf. or forced migra*.tw,kf. or stateless person*.tw,kf. or asylum seek*.tw,kf.<br>2-exp Practice Guidelines as Topic/ or exp Standard of Care/ or exp Community Health Services/ or exp Delivery of Health Care/ or exp Social Support/ or exp Mental Health Services/ or best practice*.tw,kf. or guideline*.tw,kf. or clinical pathway*.tw,kf. or psychosocial support.tw,kf. or care model*.tw,kf. or support service*.tw,kf. or community-based.tw,kf. or informal support*.tw,kf. or formal support*.tw,kf. or clinical care.tw,kf.<br>3-exp Housing/ or exp Host Family/ or homestay*.tw,kf. or "home stay*".tw,kf. or host famil*.tw,kf. or "hosted accommodation*".tw,kf. or private accommodat*.tw,kf. or "private accommodation*".tw,kf. or "community housing".tw,kf. or "non-camp setting*".tw,kf. or "out-of-camp setting*".tw,kf. or private host*.tw,kf. or community host*.tw,kf. or private housing.tw,kf. or private home*.tw,kf. or "private sponsor*".tw,kf. or "sponsor* family".tw,kf. or "community sponsor*".tw,kf.<br>4-1 and 2 and 3<br>5- limit 4 to (English language and yr="2000 -Current")
Web of Science (Clarivate)	TS=(refugee* OR "asylum seeker*" OR "displaced famil*" OR "displaced person*" OR "forced migra*" OR "stateless person*" OR "asylum seek*") AND TS=("best practice*" OR guideline* OR "clinical pathway*" OR "psychosocial support" OR "care model*" OR "support service*" OR "community-based" OR "informal support*" OR "formal support*" OR "clinical care") AND TS=(homestay* OR "home stay*" OR "host famil*" OR "hosted accommodation*" OR "private accommodat*" OR "private accommodation*" OR "community housing" OR "non-camp setting*" OR "out-of-camp setting*" OR "private host*" OR "community host*" OR "private housing" OR "private home*" OR "private sponsor*" OR "sponsor* family" OR "community sponsor*")
Scopus	(TITLE-ABS-KEY ("refugee*" OR "asylum seeker*" OR "displaced famil*" OR "displaced person*" OR "forced migra*" OR "stateless person*" OR "asylum seek*") AND TITLE-ABS-KEY ("best practice*" OR "guideline*" OR "clinical pathway*" OR "psychosocial support" OR "care

	model*" OR "support service*" OR "community-based" OR "informal support*" OR "formal support*" OR "clinical care") AND TITLE-ABS-KEY ("homestay*" OR "home stay*" OR "host famil*" OR "hosted accommodation*" OR "private accommodat*" OR "private accommodation*" OR "community housing" OR "non-camp setting*" OR "out-of-camp setting*" OR "private host*" OR "community host*" OR "private housing" OR "private home*" OR "private sponsor*" OR "sponsor* family" OR "community sponsor*")) AND PUBYEAR > 2021 AND (LIMIT-TO (LANGUAGE , "English"))
SocINDEX (via EBSCOhost)	TI (refugee* OR "asylum seeker*" OR "displaced famil*" OR "displaced person*" OR "forced migra*" OR "stateless person*" OR "asylum seek*")<br>OR AB (refugee* OR "asylum seeker*" OR "displaced famil*" OR "displaced person*" OR "forced migra*" OR "stateless person*" OR "asylum seek*") AND TI ("best practice*" OR guideline* OR "clinical pathway*" OR "psychosocial support" OR "care model*" OR "support service*" OR "community-based" OR "informal support*" OR "formal support*" OR "clinical care")<br>OR AB ("best practice*" OR guideline* OR "clinical pathway*" OR "psychosocial support" OR "care model*" OR "support service*" OR "community-based" OR "informal support*" OR "formal support*" OR "clinical care") AND TI (homestay* OR "home stay*" OR "host famil*" OR "hosted accommodation*" OR "private accommodat*" OR "private accommodation*" OR "community housing" OR "non-camp setting*" OR "out-of-camp setting*" OR "private host*" OR "community host*" OR "private housing" OR "private home*" OR "private sponsor*" OR "sponsor* family" OR "community sponsor*")<br>OR AB (homestay* OR "home stay*" OR "host famil*" OR "hosted accommodation*" OR "private accommodat*" OR "private accommodation*" OR "community housing" OR "non-camp setting*" OR "out-of-camp setting*" OR "private host*" OR "community host*" OR "private housing" OR "private home*" OR "private sponsor*" OR "sponsor* family" OR "community sponsor*")
ProQuest Dissertations and Theses	(refugee* OR "asylum seeker*" OR "displaced famil*" OR "displaced person*" OR "forced migra*" OR "stateless person*" OR "asylum seek*") AND ("best

	practice*" OR guideline* OR "clinical pathway*" OR "psychosocial support" OR "care model*" OR "support service*" OR "community-based" OR "informal support*" OR "formal support*" OR "clinical care") AND (homestay* OR "home stay*" OR "host famil*" OR "hosted accommodation*" OR "private accommodat*" OR "private accommodation*" OR "community housing" OR "non-camp setting*" OR "out-of-camp setting*" OR "private host*" OR "community host*" OR "private housing" OR "private home*" OR "private sponsor*" OR "sponsor* family" OR "community sponsor*")
CINAHL (via EBSCOhost)	((MH "Refugees+") OR TI (refugee* OR "asylum seeker*" OR "displaced famil*" OR "displaced person*" OR "forced migra*" OR "stateless person*" OR "asylum seek*") OR AB (refugee* OR "asylum seeker*" OR "displaced famil*" OR "displaced person*" OR "forced migra*" OR "stateless person*" OR "asylum seek*")) AND ((MH "Practice Guidelines") OR (MH "Standard of Care") OR (MH "Community Health Services+") OR (MH "Health Care Delivery") OR (MH "Social Support") OR (MH "Mental Health Services+") OR TI ("best practice*" OR guideline* OR "clinical pathway*" OR "psychosocial support" OR "care model*" OR "support service*" OR "community-based" OR "informal support*" OR "formal support*" OR "clinical care") OR AB ("best practice*" OR guideline* OR "clinical pathway*" OR "psychosocial support" OR "care model*" OR "support service*" OR "community-based" OR "informal support*" OR "formal support*" OR "clinical care")) AND ((MH "Housing+") OR TI (homestay* OR "home stay*" OR "host famil*" OR "hosted accommodation*" OR "private accommodat*" OR "private accommodation*" OR "community housing" OR "non-camp setting*" OR "out-of-camp setting*" OR "private host*" OR "community host*" OR "private housing" OR "private home*" OR "private sponsor*" OR "sponsor* family" OR "community sponsor*") OR AB (homestay* OR "home stay*" OR "host famil*" OR "hosted accommodation*" OR "private accommodat*" OR "private accommodation*" OR "community housing" OR "non-camp setting*" OR "out-of-camp setting*" OR "private host*" OR "community host*" OR "private housing" OR "private home*" OR "private sponsor*" OR "sponsor* family" OR "community sponsor*"))
